# Everybody's Page

**Published:** 1888-09-29

**Authors:** 


### Everybody's Page.

Lean beef, which we consider the type of solid food, contains between 70 and 80 parts of water in 100.

Some years ago, when chicory-root was dear, one firm is reported to have washed and ground some 700 tons of carrots and 350 tons of parsnips to use for the adulteration of chicory, and thus, of course, indirectly of coffee.

The air, on entering the chest, contains, as we have seen, 21 per cent. of oxygen and 4 parts in 10,000 of carbonic acid gas; expired air, on the other hand, contains only 13 per cent. of oxygen and 500 parts in 10,000 of carbonic acid gas.

Popular prejudice in favour of a white loaf has caused the use of alum in this country, whilst on the Continent the public are occasionally treated to an additional dainty in the form of sulphate of copper, better known as blue vitrol.

Dr. Worms, of the Paris Academy of Medicine, has ascertained that bees, ants, and wasps show a marked dislike to the new saccharine. To the human palate there is a difference in the taste between it and sugar. It has been shown, however, that its use disturbs digestion.

Many people do not know how easily they can protect themselves and their children against the bites of gnats and other insects. Weak carbolic acid sponged on the skin and hair, and in some cases the clothing, will drive away the whole tribe.

Thymol is contained in abundance in the volatile oil of *Monarda punctata*, the American horse-mint, which may hereafter become an important commercial source of this substance. The use of thymol as an antiseptic in dentifrices, etc., and as a general disinfectant, is rapidly increasing.

Dr. Parkes says that a labouring man, by ringing the changes on oatmeal, maize, peas and beans, rice and macaroni (which is made from corn), to which may be added cheese and bacon occasionally, may bring up his children as well nourished as those of the richest people, and at a small cost.

There is an insidious form of dust which is liable to attack our lungs, that, namely, which consists of infectious material, the low forms of growth which were described to you here a week or two ago. The germs of several diseases are believed to reach the body by being inhaled. Among these are measles, whooping-cough, and, especially, consumption. Against such we can only guard ourselves by avoiding the near neighbourhood of infection, and by keeping the lungs in as perfect health as possible.

It has been calculated that as many as 3,528 sweat-glands exist on a square inch of the surface of the palm of the hand; and since every tube, when straightened, is a quarter of an inch in length, it follows that in such a square inch there exists a length of tube equal to 882 inches, or 73½ feet. Further calculation shows that the total number of pores of the body may be about 7,000,000, and the length of the perspiratory tubing about 1,570,000 inches, or nearly twenty-eight miles.

According to recent experiments of MM. Hauriot and Richet, of which an account has been given to the French Academy of Sciences, the ventilation of the lungs is increased by muscular labour. In moderate work the ventilation is more than sufficient for the excretion of the carbonic acid produced, and above all for the absorption of the necessary oxygen. In hard work the proportions of carbolic acid produced and oxygen absorbed rise slightly the harder the work.

Ill-regulated feeding, neglect of the children's well-doing through carelessness or indifference on the part of their mothers, drugging them with opiates to keep them quiet, and entrusting them to incompetent nurses—all these things bring about fearful ravages, and are largely answerable for the high mortality of infants. Drunkenness, directly or indirectly, more than any other cause, brings about this great wrong done to helpless innocents. Feebly-born children, who come into the world only to meet with an early death, owe their sad condition but too often to drunkenness and other shameful vices on the part of parents.

Pliny says the Gauls invented the art of soap-making, and that the Romans acquired it from the Gauls and introduced it into Italy. Soap factories are known to have existed in Italy and Spain in the eighth century, in France about the thirteenth century, and in Great Britain about the fourteenth century. The first English patent regarding soap was granted in 1622 to Jones and Palmer for "the misterie, arte, way, and means of makinge of hard soape," . . . and "the arte, misterie, way, and means of makinge of softe soape." The strict science of soap-making, however, was not known till 1811, when the subject was wrought out by Chevreul. In modern times the amount of soap per head of a nation may be taken as evidence of its advance in civilization, and at the same time affords practical proof that cleanliness is next to godliness.

Lord Kames remarks, contemplating the comparatively modest requirements of London a century ago: "The annual supply amounts probably to a greater number than were needed annually for recruiting our armies and navies in the late war with France. If so, London is a greater enemy to population than a bloody war would be, supposing it even to be perpetual. What an enormous tax is Great Britain thus subjected to for supporting her capital! The rearing and educating yearly for London 7,000 or 8,000 persons requires an immense sum." In 1865 Dr. Morgan, in a paper on "The Deterioration of Race in Large Cities," estimated that in order to maintain the growth of London, "the whole available resources of a vast country nursery, peopled by nearly two millions, must be called into requisition."

Hay-Fever.—Hay fever is a nervous affection so closely analogous to asthma that it has sometimes been called "hay-asthma." It is known only among the educated classes, whose nervous systems are highly-developed; and many doctors consider it so hopeless of cure that they counsel the sufferers to go to the seaside, take a trip on a yacht, or bury themselves in a densely-populated town, so as to remove themselves from the exciting causes. Dust, draughts, the smell of hay, grass, the pollen of flowers, or even the odour of fruit will excite an attack in persons disposed to the complaint; and these have mostly no comfort but the assurance that the disease is not fatal, and that in England rain and bad weather, which bring with them immunity, are never for long strangers to us. One of the best remedies is tobacco-smoking, the smoke being retained in the mouth as long as possible and ejected through the nose. In some cases great benefit is said to have been derived from taking ten drops of tincture of nux-vomica in half a tumblerful of water. Another vaunted remedy is from three to five drops of arsenic solution—"liquor arsenicalis" of the Pharmacopœia—in a little water three times a day after meals. During a paroxysm, ten drops of simple tincture of lobelia is often given in water every quarter of an hour. The inhalation of the steam of ten drops of creosote in a pint of hot water is said to be good, or twenty drops of spirits of camphor may be added to the same quantity of water to make an inhalation. General tonic measures and nourishing diet are always advisable.

